# Beliefs about Childhood Vaccination in the United States: Political Ideology, False Consensus, and the Illusion of Uniqueness

**DOI:** 10.1371/journal.pone.0158382

**Published:** 2016-07-08

**Authors:** Mitchell Rabinowitz, Lauren Latella, Chadly Stern, John T. Jost

**Affiliations:** 1 Graduate School of Education, Fordham University, New York, New York, United States of America; 2 Department of Psychology, University of Illinois, Urbana-Champaign, Illinois, United States of America; 3 Department of Psychology, New York University, New York, New York, United States of America; University of Hyderabad, INDIA

## Abstract

Several contagious diseases were nearly eradicated through childhood vaccination, but some parents have decided in recent years not to fully vaccinate their children, raising new public health concerns. The question of whether and how beliefs about vaccination are linked to political ideology has been hotly debated. This study investigates the effects of ideology on perceptions of harms and benefits related to vaccination as well as judgments of others’ attitudes. A total of 367 U.S. adults (131 men, 236 women; *M*_age_ = 34.92 years, range = 18–72) completed an online survey through Mechanical Turk. Results revealed that liberals were significantly more likely to endorse pro-vaccination statements and to regard them as “facts” (rather than “beliefs”), in comparison with moderates and conservatives. Whereas conservatives overestimated the proportion of like-minded others who agreed with them, liberals underestimated the proportion of others who agreed with them. That is, conservatives exhibited the “truly false consensus effect,” whereas liberals exhibited an “illusion of uniqueness” with respect to beliefs about vaccination. Conservative and moderate parents in this sample were less likely than liberals to report having fully vaccinated their children prior to the age of two. A clear limitation of this study is that the sample is not representative of the U.S. population. Nevertheless, a recognition of ideological sources of potential variability in health-related beliefs and perceptions is a prerequisite for the design of effective forms of public communication.

## Introduction

Not so long ago, contagious diseases such as measles, diphtheria, and whooping cough posed serious public health threats, especially in highly populated urban environments. Thanks to scientific breakthroughs and effective campaigns to promote childhood vaccination, these diseases were nearly eradicated in the Western hemisphere by the late 1990s [1]. Since that time, however, a plurality of citizens in the United States has decided that childhood vaccination is either harmful or unnecessary [2]. As a result, some parents have stopped vaccinating their children, and the diseases have returned, raising new public health concerns [3]. In some European countries, vaccination rates have also dropped in recent years, leading to dangerous outbreaks of measles and other communicable diseases [4].

In the U.S. today, 15% of children are under-immunized because their parents hold skeptical attitudes about the safety and utility of vaccinations [2]. Surveys suggest that 20–25% of parents believe that children receive too many vaccinations prior to the age of two, and 29–33% of parents believe—in contrast to the scientific consensus—that having multiple vaccines at a young age can weaken a child’s immune system [2, 5]. Parents who oppose vaccinations often doubt that vaccines protect against serious diseases, believe that their child’s immune system can protect itself against diseases without vaccines, and claim that the side effects of vaccination may be more harmful than the disease itself [2, 5].

By contrast, parents who support vaccinations are more likely to perceive childhood susceptibility to serious, vaccine-preventable diseases and to anticipate feeling regret if their children were to become infected with these diseases [5]. Whereas parents who are skeptical about the effectiveness and safety of vaccinations are more influenced by their peers than by medical experts, parents who support vaccinations are more likely to trust their pediatricians’ recommendations [5, 6]. Not surprisingly, there are also demographic factors such as socio-economic status, race, education, and size of household that are correlated with decisions about whether or not to vaccinate one’s children [2, 5, 7]. In any case, a significant gap has emerged between experts and the general public when it comes to the issue: whereas 82% of scientists believed in 2009 that childhood vaccination should be mandatory in the U.S., only 69% of survey respondents from the broader population agreed [8].

This brief discussion highlights the fact that parents are sensitive to the perceived harms and benefits of vaccinating their children and that there is considerable variability in these perceptions [9, 10]. The decision about whether to vaccinate one’s children appears to be based less on an attempt to understand the scientific consensus than on personalized estimates of risks and benefits. Because this is often the case when it comes to health-related decisions, research in health psychology frequently focuses on communicative attempts to modify individuals’ beliefs about health-related practices [9, 11]. Before designing effective interventions, however, it is necessary to understand whether there is systematic variability in perceptions of risks and benefits in different social groups [12, 13].

The current study investigates the possible role of ideological differences in accounting for variability in beliefs about childhood vaccination. Unfortunately, we were unable to examine this possibility in the context of a nationally representative survey of the general population. Instead, we made use of a convenience sample of Mechanical Turk workers. As a result, it is not possible to generalize our findings to the U.S. population as a whole. Nevertheless, this study provides some of the first systematic evidence that there are meaningful connections between political attitudes, on one hand, and scientific beliefs and perceptions of social norms concerning childhood vaccination, on the other.

### The Role of Political Ideology

It would appear that reluctance to vaccinate one’s children is connected to a number of specific beliefs that have circulated in popular culture [14]. In addition to various types of social pressures [15], receptivity to skeptical beliefs may be linked to underlying motivational orientations, such as distrust of scientific or governmental authorities as well as corporate actors, such as pharmaceutical companies and health insurance agencies. Taken in conjunction, these considerations suggest that certain motivated belief systems, such as religious [4] and political ideologies [16], may exaggerate—or, in other cases, attenuate—skepticism about the importance of vaccinating children.

Political ideology could affect beliefs about vaccination in multiple, perhaps conflicting ways. Because there are prominent skeptics on the cultural left (e.g., Robert F. Kennedy Jr., Bill Maher, and Jenny McCarthy), it has been suggested that the anti-vaccination movement in the U.S. is largely a liberal development—both in terms of the movement’s origins and the people who should be most susceptible to messages that are critical of the “medical-industrial complex.” The assumption, therefore, is that liberals would be especially likely to perceive vaccines as harmful or unnecessary [17]. Along these lines, an editorial in the *National Review* stated that, “The countercultural Left is and has always been the core of the anti-vaccination movement. From NPR to PBS to the broadcast networks, the message of all-natural food and holistic, homeopathic medicine has been broadcast for decades, while traditional scientific medicine has been brought under suspicion as something being put over on us by the government and corporations” [18]. Generally (but not precisely) consistent with this logic, the states that have granted the most exemptions from vaccinating kindergarteners are somewhat more likely to be liberal-leaning states (such as Oregon, Illinois, Vermont, Michigan, and Maine) than conservative-leaning states—although some of the latter (such as Idaho, Alaska, Arizona, and Utah) also have fairly high rates of exemptions [19, 20]. It should be noted however, that it is impossible to draw unambiguous conclusions about individual behavior on the bases of aggregate (e.g., state-level) data because of “Simpson’s paradox,” which demonstrates that trends at the individual level may be very different from aggregate-level trends; e.g., [20]. For instance, it is conceivable that statewide trends in vaccination are driven by conservatives in “blue” states or liberals in “red” states.

On the other hand, there are reasons to suppose that resistance to vaccination would be stronger on the political right. On many issues, such as evolution, climate change, stem cell research, and AIDS prevention, conservatives are less likely to trust scientific recommendations than are liberals; e.g., [21, 22]. There are probably several reasons for this, including dis-identification with scientists and educational elites [23], identification with religious authorities [21], and cognitive-motivational styles that favor intuitive, heuristic-driven ways of thinking over more systematic, deliberative modes of processing information [24]. Furthermore, libertarian opposition to governmental mandates may lead economic conservatives to be more supportive of parental decisions to refrain from vaccinating their children. For Mooney [25], Republican Senator Rand Paul’s declaration that vaccinations cause “profound mental disorders” was enough to puncture the myth “that denying vaccine safety, or avoiding vaccinations, is a liberal, granola thing.”

Finally, a public opinion poll of 1,000 U.S. adults administered by YouGov and the Huffington Post revealed that—despite much ideological bickering among politicians and pundits—there are no clear stereotypes linking one group of ideological adherents more strongly than others to skepticism about vaccination [26]. When respondents were asked, “Which group do you think is MORE likely to have doubts about childhood vaccinations?” the results were equivocal at best. Twenty-six percent cited “Liberals” and 26% cited “Conservatives,” with 27% saying they were “Unsure” and 21% saying “Neither.” When these results were broken down according to respondents’ own ideological orientation, they revealed that conservatives assumed that liberals were more dubious about vaccination than conservatives, whereas liberals assumed that conservatives were more dubious than liberals [26].

What is the *actual* nature of the relationship, if any, between political ideology and beliefs about vaccination? Here, too, the evidence is ambiguous. A 2009 Pew Survey suggested that 71% of Democrats and 71% of Republicans felt that childhood vaccinations should be required [8]. Another poll conducted the same year by USA Today/Gallup focused on whether people had been influenced by actress Jenny McCarthy’s claims that her son had developed autism because of vaccinations. Results suggested that liberals were slightly more aware of McCarthy’s statements than were conservatives, but they were also slightly less likely than conservatives to report having been *persuaded* by those comments. A 2015 poll found that 61% of Democrats and 62% of Republicans believed that “The science supporting the safety of childhood vaccination is indisputable” [26], but other studies suggest that Democrats are more accepting than Republicans of scientific recommendations concerning childhood vaccination as well as many other health-related issues; e.g., [21]. A survey conducted by Kahan [27] suggested that conservative Republicans perceived the risks of vaccination to be greater and the benefits to be lesser, in comparison with liberal Democrats. Few, if any, studies of public opinion have delved more deeply into the specific contents of beliefs about vaccination and whether there are consistent ideological differences in these contents.

### The Fact/Belief Distinction, False Consensus, and the Illusion of Uniqueness

In addition to the obvious question of whether people agree or disagree with various statements about vaccination, it is useful to assess perceptions about whether the statements ought to be categorized as “facts” or “beliefs.” There is a long history of discussion in philosophy [28, 29] and psychology [30, 31] about how to characterize propositional statements as facts or beliefs. In these discussions, it is generally assumed that a fact is a concept from classical logic that has a set of defining features. At the same time, in practice people tend to treat facts and beliefs as “fuzzy concepts,” insofar as the features they use to distinguish facts from beliefs vary from one domain to another [32]. People tend to categorize information as factual when they believe that nearly everyone else agrees with it; cf. [33]. Therefore, exploring the ways in which people categorize specific statements about vaccination as facts versus beliefs enables us to go beyond the mere assessment of personal agreement and to incorporate perceptions of social norms.

Furthermore, we are able to investigate directly individuals’ perceptions of how widespread (or consensually shared) certain beliefs about vaccination are both within and between ideological groups. Past research conducted in non-health-related domains suggests that conservatives are more likely than liberals to assume that likeminded others (and people in general) share their own beliefs [34]. Liberals and conservatives also seem to differ when it comes to the *accuracy* of their consensus estimates. Conservatives may be prone to *overestimate* the extent to which likeminded others share their beliefs (i.e., judging their beliefs to be more common than they actually are), thereby exhibiting what has been termed the “truly false consensus effect” [35, 36]. By contrast, liberals may be prone to *underestimate* the extent to which likeminded others share their beliefs (i.e., judging their beliefs to more distinctive than they actually are), thereby exhibiting “an illusion of uniqueness” [37].

Ideological differences in perceptions of consensus are potentially consequential when it comes to health-related decisions, insofar as human behavior is strongly affected by social norms, that is, perceptions about whether other people (especially likeminded others) are engaging in a given behavior [38]. For example, research in health psychology has demonstrated that the perceived prevalence of harmful behaviors—such as smoking tobacco, binge drinking, and illicit drug usage—affects the likelihood that a person will engage in that behavior, and that people frequently use these perceptions to justify their behavior [39–42]. Thus, the perceived prevalence of one’s own attitudes about vaccination and the extent to which those perceptions are accurate may have broad-based implications for personal and public health outcomes.

### Overview of the Current Research

In the present research program, we addressed two general questions about the relationship between political ideology and beliefs about childhood vaccination. First, we examined whether liberals, moderates, and conservatives would differ in their perceptions of scientific information about vaccination. In particular, we sought to determine whether ideology would be related to (a) the likelihood of endorsing pro- and anti-vaccination statements, and (b) the likelihood of categorizing these statements as facts versus beliefs. Second, we investigated whether ideology was related to actual and perceived rates of consensus with respect to beliefs about vaccination. Specifically, we determined the extent to which liberals, moderates, and conservatives (a) possessed consensus in their opinions about vaccination within their ideological group, (b) *perceived* likeminded and non-likeminded others to share their beliefs about vaccination information, and (c) *accurately* perceived the prevalence of their opinions.

## Method

### Participants

We collected data from two non-representative samples of participants using the Mechanical Turk website (see [43], for a discussion of this platform as a research tool), which in total consisted of three hundred and sixty-seven U.S. adults (131 men, 236 women; *M*_age_ = 34.92 years, range = 18–72). Our initial sample size was 197, but reviewers asked us to supplement the sample with additional participants. We therefore added data obtained in the same manner from 170 other participants who completed the entire survey. We did not include data from a subset of participants in the second set of data collection because they were assigned to complete the items in a different order. In total, 405 individuals (from both samples) started the survey as described here, but only 376 people completed it, including the measure of political orientation, which was at the end of the survey. Nine recruits were excluded from statistical analysis because of suspicious or abnormal response patterns. Eight of them agreed with every statement, and one had a disagreement rate that was more than 3 standard deviations from the sample mean. Of these participants, 282 identified themselves as White (76.8%), twenty-six as Asian (7.1%), twenty-four as Black or African American (6.5%), nineteen as Hispanic or Latino (5.2%), five as Native American or Pacific Islander (1.4%), and eleven as “Other” (3%). The sample that participants came from did not significantly moderate any of the results, so this variable is not discussed further.

The request for participants, methodology, materials and procedures used to insure anonymity were reviewed and approved by the Fordham University IRB board, # IRB-14-12-MR-101. On the first page of the survey, a consent form was presented. Participants were informed that if they proceeded to the next page then consent would be assumed. Prior to analyzing the data, all information that could identify the participant was removed from the data set.

### Materials and Procedure

Participants first provided demographic information, including information about their own political ideology. To obtain a categorical measure of ideology [37, 44], we asked participants to indicate which of the following descriptions best applied to them: “Liberal,” “Moderate,” or “Conservative.” Our final sample was comprised of 158 liberals (43.1%), 126 moderates (34.3%), and 83 conservatives (22.6%).

#### General statements

To familiarize themselves with the fact/belief categorization task, participants were presented with 20 general statements used in prior research [32], including 10 statements that were generally accepted as facts (e.g., “The earth revolves around the sun”) and 10 that were beliefs (e.g., “Sleeping with the windows open is good for you”). For each statement, participants were asked the following questions: (1) “How strongly do you agree with this statement?” (4-point Likert scale: 1 = “strongly disagree,” 4 = “strongly agree”); (2) “What percentage of the general adult population would agree with this statement?” (5-point scale: 0%, 25%, 50%, 75%, or 100%); and (3) “Is this statement a fact or belief?” (dichotomous response). To ensure that participants were taking the study seriously, we confirmed that their responses to general statements were similar to those obtained in prior research [30].

#### Vaccination statements

Next, participants completed a similar set of questions with respect to 20 statements pertaining to childhood vaccination, including 10 pro-vaccination and 10 anti-vaccination statements, presented in random order (see S1 Appendix). Statements were derived from websites and media sources, including vaccines.procon.org, which featured a compilation of pro- and anti-vaccination statements based on research from the Center for Disease Control, as well as information from parents.com, kidshealth.org, huffingtonpost.com, and NYTimes.com. Additional statements were based on information provided by Harvard Health Publications, the U.S. Department of Health and Human Services website, thehealthyhomeeconomist.com, and DrFeder.com (a homeopathic pediatrician's website). A sample pro-vaccination statement is: “Vaccinations mobilize antibodies and proteins that are mimicked in the body’s natural immune defenses.” A sample anti-vaccination statement is: “The increased number of vaccinations prior to a child’s second birthday is the reason why there has been an increase in Autism Spectrum Disorder in children.”

For each of the 20 vaccination statements, participants were asked the following five questions: (1) “How strongly do you agree with this statement?” (1 = “strongly disagree,” 4 = “strongly agree”) (2) “What percentage of the general adult population would agree with this statement?” (3) “What percentage of liberals within the U.S. would agree with this statement?”; (4) “What percentage of conservatives within the U.S. would agree with this statement?” (Responses to Questions 2–4 were made use of 5-point scales: 0%, 25%, 50%, 75%, or 100%); and (5) “Is this statement a fact or belief?” (dichotomous response).

#### Childhood vaccination decisions

At the end of the survey participants answered two questions about their families: “Do you have children?”; and “Have you fully vaccinated your children before the age of two?”

## Results

### Endorsement of Pro- vs. Anti-Vaccination Statements

We first investigated whether liberals and conservatives differed in their endorsement of pro- vs. anti-vaccination statements. Participants provided responses to 20 statements. Nonindependence in participants’ responses can result in biased standard error estimates and, in turn, significance testing [45]. To account for nonindependence in participants’ responses, we conducted a model using generalized estimating equations (GEE). When using GEE researchers must specify the type of working correlation matrix they wish to model. We had no theoretical reason to expect that the variances and covariances of the residuals would differ systematically across items. Therefore, we specified an exchangeable correlation matrix, which assumes that responses within participants are equally correlated. The political ideology variable contained three levels, and so we created two dummy-coded contrast variables for analyses [46]. We selected liberals as the reference group. One dummy contrast compared liberals vs. moderates, and the other compared liberals vs. conservatives. Predictors in the model were statement type (-1 = Anti-vaccination, 1 = Pro-vaccination), the liberal/moderate contrast, the liberal/conservative contrast, and two-way interactions between statement type and each dummy contrast. The dependent variable was participants’ level of agreement with the item.

**Fig 1:**
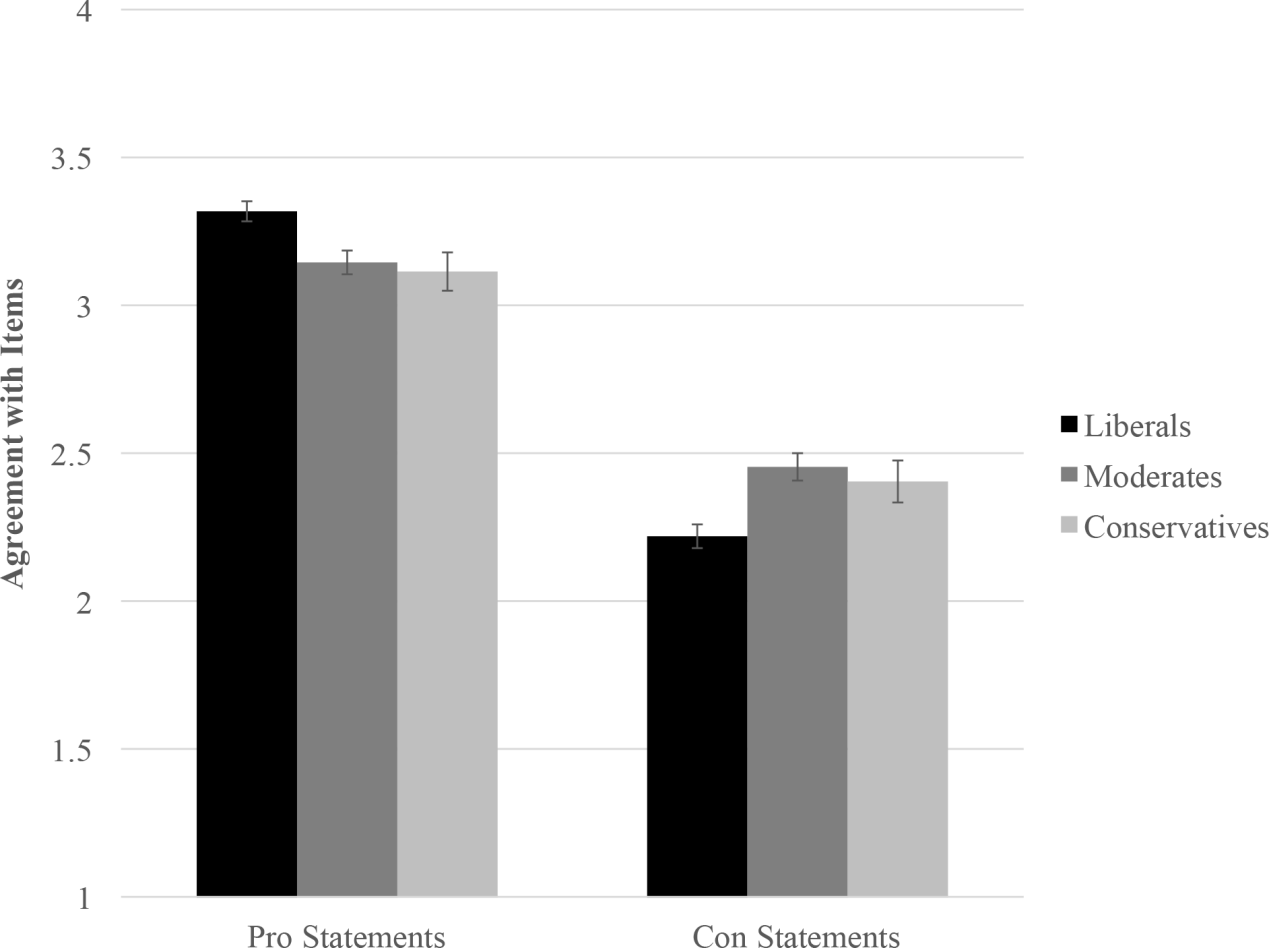
Agreement with items plotted as a function of item type and participant ideology.

The analysis yielded significant effects for the statement type × liberal/moderate contrast, *B* = -.21, *SE* = .05, *z* = -3.84, *p* < .001, 95% CI [-.31, -.10], and the statement type × liberal/conservative contrast, *B* = -.20, *SE* = .07, *z* = -2.73, *p* = .006, 95% CI [-.34, -.06]. Thus, political ideology did affect the degree to which participants endorsed pro- vs. anti-vaccination statements (see Fig 1). Liberals expressed greater endorsement of pro-vaccination statements in comparison with moderates, *B* = -.17, *SE* = .05, *z* = -3.16, *p* = .002, 95% CI [-.28, -.07], and conservatives, *B* = -.20, *SE* = .07, *z* = -2.78, *p* = .006, 95% CI [-.35, -.06]. Moderates and conservatives did not differ from one another in their endorsement of pro-vaccination statements, *B* = -.03, *SE* = .08, *z* = -.38, *p* = .71, 95% CI [-.18, .12].

Liberals also expressed less agreement (or more disagreement) with anti-vaccination statements in comparison with moderates, *B* = .24, *SE* = .06, *z* = 3.86, *p* < .001, 95% CI [.12, .36], and conservatives, *B* = .19, *SE* = .08, *z* = 2.30, *p* = .02, 95% CI [.03, .35]. Moderates and conservatives did not differ from one another in their endorsement of anti-vaccination statements, *B* = -.05, *SE* = .09, *z* = -.60, *p* = .55, 95% CI [-.22, .12]. Liberals (*B* = .55, *SE* = .03, *z* = 16.13, *p* < .001, 95% CI [.48, .62]), moderates (*B* = .34, *SE* = .04, *z* = 8.31, *p* < .001, 95% CI [.26, .43]), and conservatives (*B* = .36, *SE* = .06, *z* = 5.66, *p* < .001, 95% CI [.23, .48]) were all more likely to endorse pro- than anti-vaccination statements.

### Categorization of Facts versus Beliefs

Next we assessed whether liberals, moderates, and conservatives differed in the extent to which they categorized pro- and anti-vaccination statements as “facts” (versus “beliefs”). To account for the non-independence in participants’ responses to the 20 statements, we conducted another model using GEE. Because participants indicated whether each statement was a fact or belief in a dichotomous manner, we specified a binary logistic outcome with an exchangeable correlation matrix [47]. Predictors in the model were statement type (-1 = Anti-vaccination, 1 = Pro-vaccination), the liberal/moderate contrast, the liberal/conservative contrast, and two-way interactions between statement type and each dummy contrast. The dependent variable was whether participants judged the statement to be a fact or belief.

**Fig 2:**
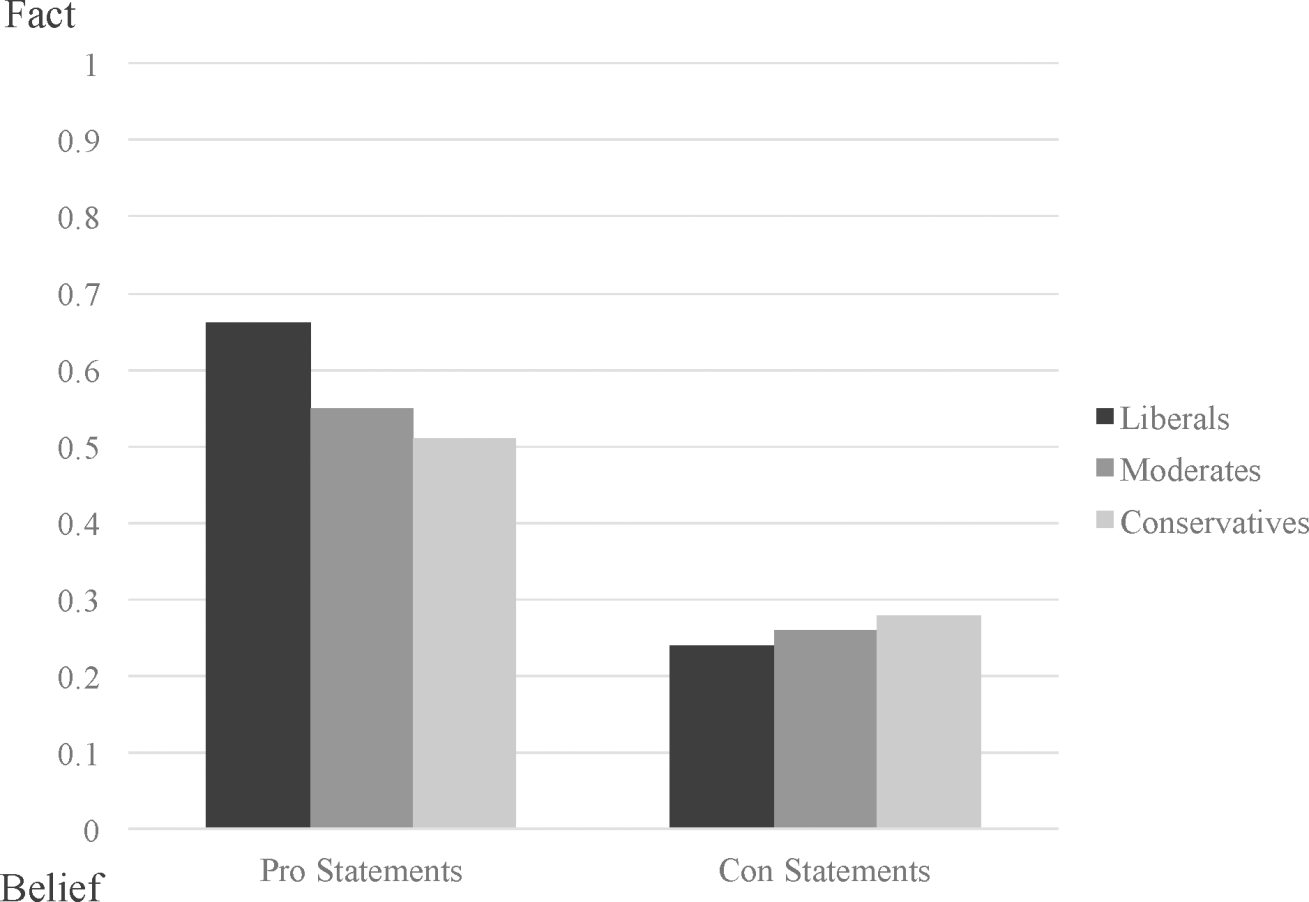
Judgments of fact vs. belief plotted as a function of item type and participant ideology.

Once again, both the statement type × liberal/moderate contrast, *B* = -.27, *SE* = .09, *z* = -3.11, *p* = .002, 95% CI [-.44, -.10], and the statement type × liberal/conservative contrast were significant, *B* = -.42, *SE* = .10, *z* = -4.07, *p* < .001, 95% CI [-.62, -.22], indicating that ideology affected the likelihood that pro- vs. anti-vaccination statements would be perceived as facts rather than beliefs (see Fig 2). Liberals were more likely to state that pro-vaccination statements were facts (vs. beliefs), in comparison with moderates, *B* = -.43, *SE* = .15, *z* = -2.78, *p* = .005, 95% CI [-.73, -.13], and conservatives, *B* = -.62, *SE* = .17, *z* = -3.61, *p* < .001, 95% CI [-.96, -.28]. Moderates and conservatives did not differ significantly from one another in the extent to which they judged pro-vaccination statements to be facts, *B* = -.19, *SE* = .17, *z* = -1.10, *p* = .27, 95% CI [-.53, .15]. There were no ideological differences in the tendency to regard anti-vaccination statements as facts vs. beliefs (liberal vs. moderate: *B* = .11, *SE* = .13, *z* = .89, *p* = .37, 95% CI [-.13, .36]; liberal vs. conservative: *B* = .21, *SE* = .16, *z* = 1.36, *p* = .17, 95% CI [-.09, .52]; moderate vs. conservative: *B* = .10, *SE* = .17, *z* = .61, *p* = .54, 95% CI [-.22, .43]). Liberals (*B* = .89, *SE* = .06, *z* = 14.50, *p* < .001, 95% CI [.77, 1.01]), moderates (*B* = .62, *SE* = .06, *z* = 10.13, *p* < .001, 95% CI [.50, .74]), and conservatives (*B* = .48, *SE* = .08, *z* = 5.81, *p* < .001, 95% CI [.32, .64]) were all more likely to judge pro-vaccination statements as facts, in comparison with anti-vaccination statements.

### Actual and Perceived Consensus: Endorsement of Pro- vs. Anti-Vaccination Statements

We assessed the amount of actual consensus in endorsement of pro- vs. anti-vaccination statements using the Social Relations Model (SRM) [48]. The SRM is a componential analysis that partitions the variance in responses into three sources: *target variance*, which measures agreement among participants; *perceiver variance*, which measures individual differences in response bias; and *error variance*, which measures residual variance. We were most interested in target variance because it captures the degree of actual consensus in responses. To examine whether participants possessed consensus in their responses, we tested whether absolute target variances were significantly greater than zero. We report the absolute variances, standard errors of the absolute variances, Wald *z*s, and *p-*values for these analyses in the text. To examine the magnitude of consensus, we calculated the percentage of response variance that was attributable to shared agreement. We calculated this relative variance value by dividing the absolute target variance by the total variance in responses [49]. Relative target and perceiver variances are reported in Table 1.

**Table 1:** Relative target and perceiver variances for the classification of facts vs. beliefs and attitudes toward vaccination statement as a function of participant’s ideological group.

	Facts versus Beliefs		Attitudes	
	Target variance	Perceiver variance	Target variance	Perceiver variance
Liberals	27.14**	11.73***	43.69**	1.28**
Moderates	17.89**	14.87***	26.43**	.83
Conservatives	12.97**	16.71***	21.28**	2.06*
All participants	20.24**	13.98***	32.16**	1.28***

* *p* < .05

** *p* < .01

*** *p* < .001

We conducted separate analyses for liberals, moderates, and conservatives, as well as for the three groups combined. Target variance was significantly different from zero for liberals, *var* = .44, *SE* = .14, *z* = 3.06, *p* = .002, 95% CI [.23, .83], moderates, *var* = .21, *SE* = .07, *z* = 3.02, *p* = .003, 95% CI [.11, .40], conservatives, *var* = .21, *SE* = .07, *z* = 2.95, *p* = .003, 95% CI [.11, .40], and for all three groups combined, *var* = .30, *SE* = .10, *z* = 3.07, *p* = .002, 95% CI [.16, .56]. This indicates that there was a significant amount of agreement within each ideological group and the sample as a whole with respect to beliefs about childhood vaccination. As shown in Table 1, liberals tended to agree more than moderates or conservatives, insofar as within-group similarity in responses to items was, descriptively speaking, greater for liberals than for the other two groups.

### Assumptions of Attitudinal Similarity vs. Dissimilarity

We next examined whether individuals perceived that others shared their attitudes about childhood vaccination. We created a single score for each participant assessing the extent to which they estimated that others shared their attitudes. To create this score, we calculated within-subject assumed similarity scores [50], as described in S2 Appendix. Positive scores reflect the assumption that others’ attitudes are *similar* to one’s own attitudes (i.e., assumed similarity), and negative scores indicate assuming that others’ attitudes are *different* from one’s own attitudes (i.e., assumed dissimilarity).

The initial assumed similarity score that participants receive is a correlation. However, Pearson’s *r* is bounded at 1 and -1, and so the sampling distributions of correlations become skewed as the size of the correlation increases [51]. Using a correlation as a dependent variable in analyses would in turn result in highly biased standard error estimates. To avoid this issue, we unbounded the correlations following methods established in prior research [36]. We converted within-subject assumed similarity scores to Fisher’s *z*-scores. For each participant we calculated an assumed similarity score with respect to perceptions of the general population’s attitudes, perceptions of liberals’ attitudes, and perceptions of conservatives’ attitudes. Five participants provided estimates that resulted in an undefined score (i.e., a correlation could not be calculated), and so they were excluded from this analysis.

**Fig 3:**
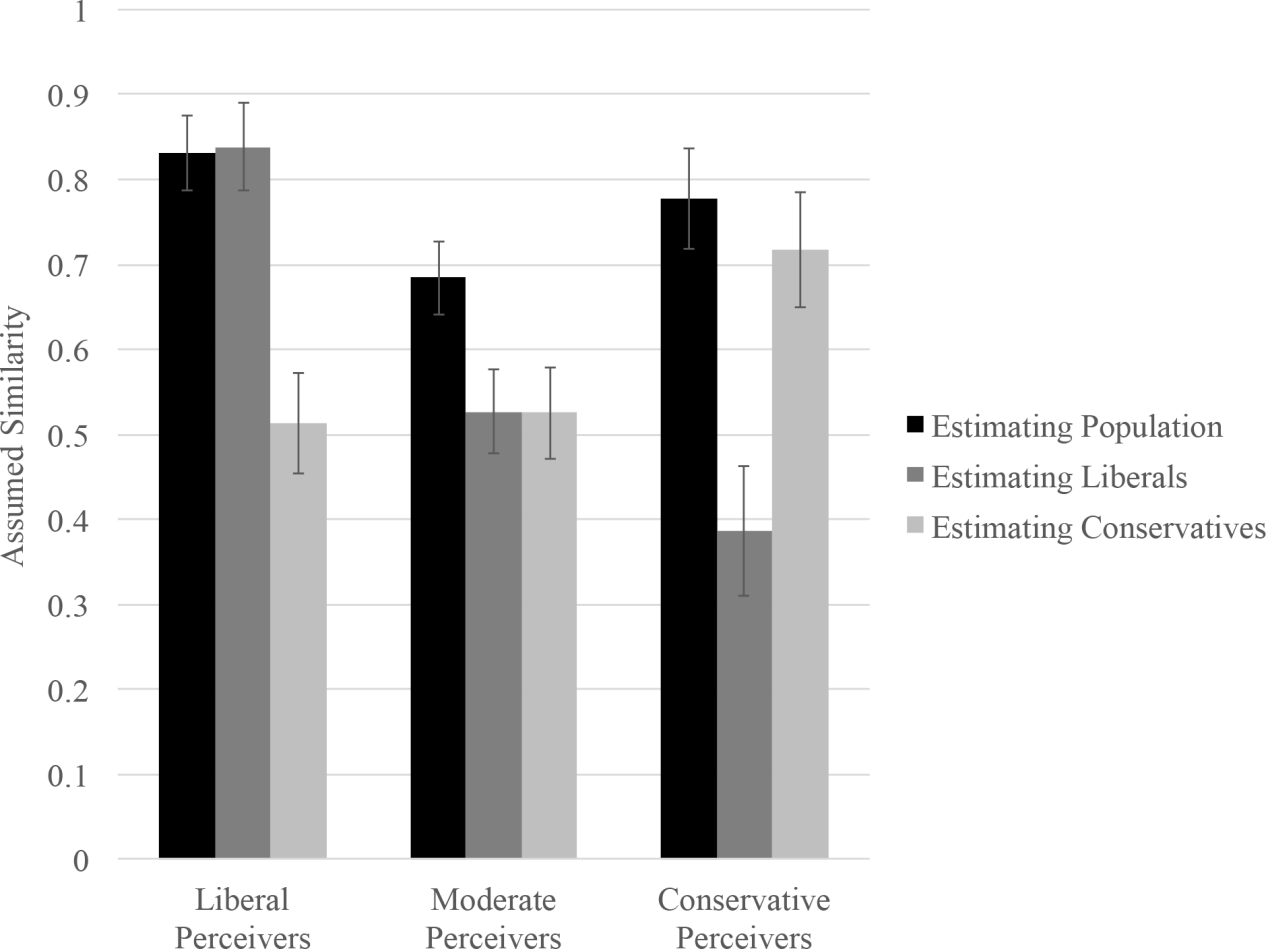
Estimates of similarity plotted as a function of participant ideology and target group.

We conducted a 3 (Participant Ideology: Liberal, Moderate, Conservative) × 3 (Comparison Group: Population, Liberals, Conservatives) repeated measures analysis of variance (RM ANOVA), with the second factor specified as repeated and the assumed similarity score as the dependent variable. We conducted an RM ANOVA for this analysis because it accounts for the repeated nature of responses across comparison groups, and all of the independent variables were categorical. Additionally, using RM ANOVA facilitates the examination of simple effects because, unlike GEE, it does not require the creation of dummy-coded contrast variables when the predictor variables contain more than two levels [52]. The ideology × comparison group interaction was significant, *F*(4, 714) = 17.89, *p* < .001, η_p_^2^ = .09 (see Fig 3). We decomposed this interaction by examining the extent to which liberals, moderates, and conservatives differentially assumed similarity between their own attitudes and those of the three comparison groups.

#### Liberals

Liberals assumed that the three groups did not share their own attitudes to the same extent, *F*(2, 356) = 35.56, *p* < .001, η_p_^2^ = .17. More specifically, liberals assumed that other liberals and members of the general population would share their own attitudes to a greater extent than conservatives would (liberal vs. conservative: *t*(356) = 5.43, *p* < .001, *d* = .40, 95% CI [.21, .44]; population vs. conservative: *t*(356) = 8.39, *p* < .001, *d* = .62, 95% CI [.24, .39]). Liberals assumed that their attitudes were shared more or less equally by other liberals and by the general population, *t*(356) = -.18, *p* = .85, *d* = .01, 95% CI [-.08, .07].

#### Moderates

Moderates also assumed that the three groups would differentially share their attitudes, *F*(2, 356) = 17.11, *p* < .001, η_p_^2^ = .09. Specifically, moderates perceived that members of the general population would be more likely to share their attitudes than would liberals, *t*(356) = 3.67, *p* < .001, *d* = .37, 95% CI [.07, .24], and conservatives, *t*(356) = 3.70, *p* < .001, *d* = .38, 95% CI [.07, .25]. Moderates did not differ in the extent to which they believed that liberals and conservatives would share their attitudes, *t*(356) = -.01, *p* = .98, *d* = .002, 95% CI [-.13, .13].

#### Conservatives

Conservatives likewise assumed that the three groups would not share their own attitudes to the same extent, *F*(2, 356) = 30.91, *p* < .001, η_p_^2^ = .15. Conservatives perceived that other conservatives and the general population would share their attitudes to a greater extent than liberals would (conservative vs. liberal: *t*(356) = 3.99, *p* < .001, *d* = .42, 95% CI [.17, .49]; population vs. liberal: *t*(356) = 7.38, *p* < .001, *d* = .77, 95% CI [.29, .50]). Conservatives assumed that their attitudes were shared more or less equally by other conservatives and by the general population, *t*(356) = 1.15, *p* = .26, *d* = .13, 95% CI [-.17, .04].

### Questions of Accuracy: Truly False Consensus vs. the Ilusion of Uniqueness?

We next examined the extent to which individuals were *accurate* in their assumptions about whether others shared their attitudes about childhood vaccination. We created a single score for each participant to determine whether they accurately estimated the extent to which others shared their attitudes. To create this score, we calculated within-subject accuracy scores [37, 50], as described in S3 Appendix. Positive scores reflect the assumption that others’ attitudes are more *similar* to one’s own attitudes than they actually are (i.e., overestimating similarity), and negative scores reflect the assumption that others’ attitudes are more *different* from one’s own attitudes than they actually are (i.e., underestimating similarity). We converted the within-subject accuracy scores to Fisher’s *z* scores so that they could be used as dependent variables in our analyses. For each participant, we calculated this score with respect to perceptions of the general population’s attitudes, perceptions of liberals’ attitudes, and perceptions of conservatives’ attitudes.

**Fig 4:**
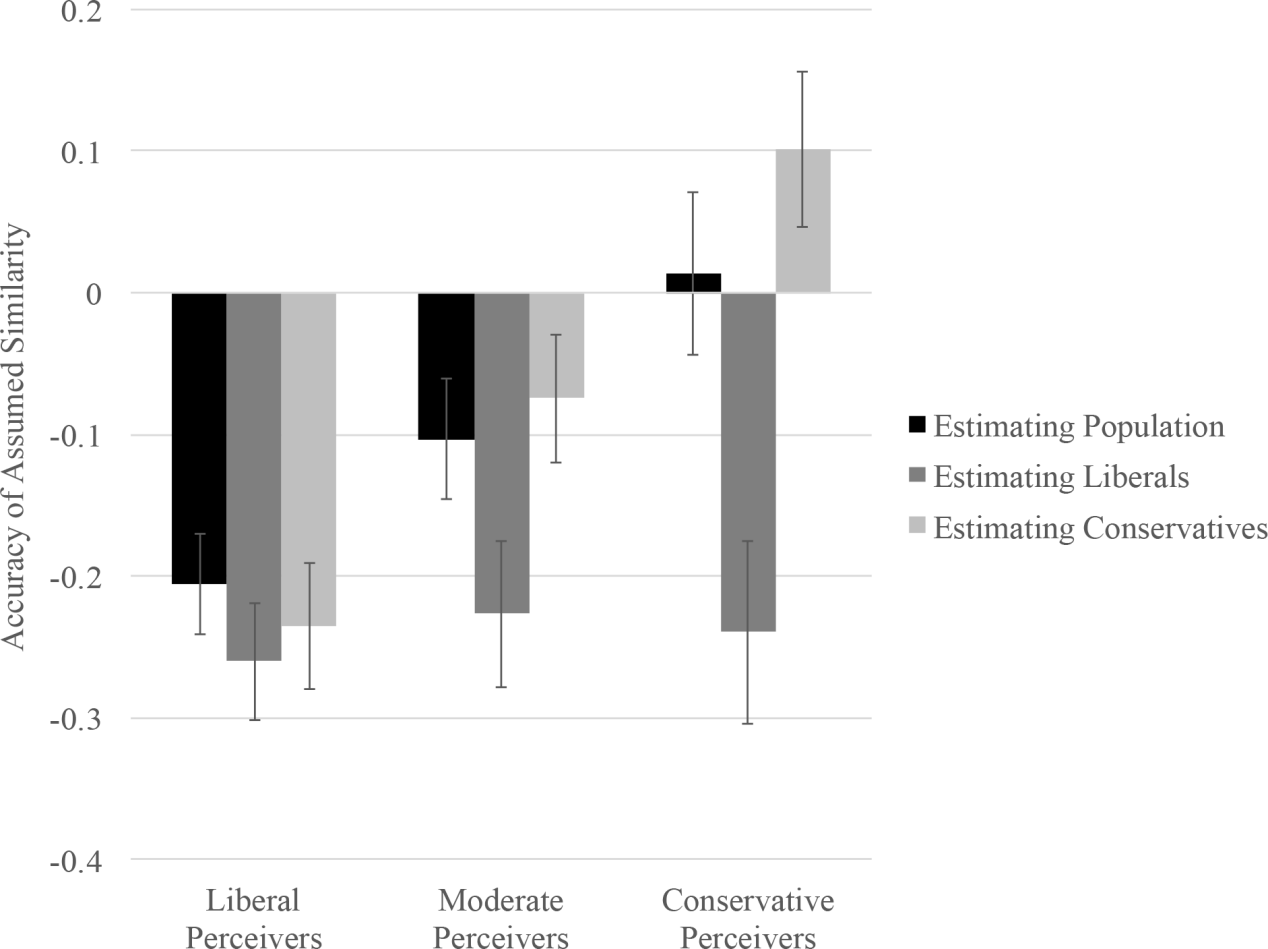
Accuracy of similarity estimates plotted as a function of participant ideology and target group.

We conducted a 3 (Participant Ideology: Liberal, Moderate, Conservative) × 3 (Comparison Group: Population, Liberals, Conservatives) RM ANOVA, with the second factor specified as repeated and accuracy score as the dependent variable. The ideology × comparison group interaction was significant, *F*(4, 728) = 7.04, *p* < .001, η_p_^2^ = .04 (see Fig 4). We decomposed this interaction by examining the extent to which liberals, moderates, and conservatives accurately perceived similarity between their own attitudes and those of the three groups. To examine accuracy for each target group, we compared accuracy scores against zero using one-sample *t*-tests [37, 50].

#### Liberals

The extent to which liberals were accurate differed as a function of comparison group, *F*(2, 363) = 3.63, *p* = .03, η_p_^2^ = .02. Liberals assumed that the attitudes of the general population, *t*(157) = -5.77, *p* < .001, *d* = .46, 95% CI [-.28, -.14], other liberals, *t*(157) = -6.35, *p* < .001, *d* = .51, 95% CI [-.34, -.18], and conservatives, *t*(157) = -5.26, *p* < .001, *d* = .42, 95% CI [-.32, -.15], were all more different from their own attitudes than was actually the case. In other words, liberals consistently underestimated the extent to which others shared their attitudes about childhood vaccination.

#### Moderates

The extent to which moderates were accurate also differed as a function of comparison group, *F*(2, 363) = 9.10, *p* < .001, η_p_^2^ = .05. Moderates underestimated how similar their own attitudes were to those of the general population, *t*(125) = -2.42, *p* = .02, *d* = .22, 95% CI [-.19, -.02], and liberals, *t*(125) = -4.41, *p* < .001, *d* = .39, 95% CI [-.33, -.12]. However, moderates did not systematically distort the differences between their own attitudes and those of conservatives, *t*(125) = -1.66, *p* = .10, *d* = .15, 95% CI [-.16, .01].

#### Conservatives

The extent to which conservatives were accurate also differed as a function of comparison group, *F*(2, 363) = 25.15, *p* < .001, η_p_^2^ = .12. Conservatives marginally *overestimated* the extent to which other conservatives shared their attitudes, *t*(82) = 1.84, *p* = .069, *d* = .20, 95% CI [-.01, .21], and significantly *underestimated* the extent to which liberals shared their own attitudes, *t*(82) = -3.72, *p* < .001, *d* = .41, 95% CI [-.37, -.11]. At the same time, they did not systematically distort differences between their own attitudes and those of the general population, *t*(82) = .24, *p* = .81, *d* = .03, 95% CI [-.10, .13].

### Actual and Perceived Consensus: Categorization of Facts vs. Beliefs

We assessed the degree of actual consensus in judgments about which statements were facts versus beliefs using the SRM, as described above. Target variance was significantly different from zero for liberals, *var* = .07, *SE* = .02, *z* = 3.04, *p* = .002, 95% CI [.04, .13], moderates, *var* = .04, *SE* = .01, *z* = 2.99, *p* = .003, 95% CI [.02, .08], conservatives, *var* = .03, *SE* = .01, *z* = 2.89, *p* = .004, 95% CI [.02, .06], and for all three groups combined, *var* = .05, *SE* = .02, *z* = 3.06, *p* = .002, 95% CI [.03, .09]. This indicates that there was a significant degree of consensus within each group and the sample as a whole about which statements were considered facts versus beliefs. As shown in Table 1, liberals exhibited more within-group consensus than did moderates or conservatives.

### Decisions about Whether to Vaccinate Their Own Children

We conducted a Chi-square analysis to determine whether liberals, moderates, and conservatives differed in terms of decisions to vaccinate their children. Only participants who reported having children were included in this analysis. The effect of ideology on whether the participant’s children were fully vaccinated or not was significant, χ^2^(2) = 6.71, *p* = .04. Conservatives and moderates were less likely than liberals to vaccinate their children. As shown in Table 2, nineteen parents indicated that they had not fully vaccinated their children. Eight of them identified as conservative, nine as moderate, and two as liberal. Thus, 17.02% of conservatives and 15.52% of moderates with children chose not to fully vaccinate their children, as compared with 3.17% of liberals. Of course, all of these ideological differences should be interpreted with caution, given that the sample was relatively small and not representative of the U.S. population as a whole.

**Table 2:** Number and percentage of participants in each ideological group who had children and did not fully vaccinate their children.

	Had Children		Did not Fully Vaccinate Children	
	Number in Ideological Group	Percent of (Ideological) Group	Number in Ideological Group	Percent of (Ideological) Group
Liberals	63	39.87%	2	3.17%
Moderates	58	46.03%	9	15.52%
Conservatives	47	56.63%	8	17.02%
All participants	168	45.78%	19	11.31%

## General Discussion

It has been hotly debated whether skepticism about childhood vaccination is more prevalent on the political left or right [17, 21, 23, 25, 27]. Previous studies were, for the most part, limited to analyses of general statements of approval or disapproval of vaccination policies, and results were inconclusive concerning the role of political ideology; e.g., [8, 26]. Our primary goal was to determine whether people’s beliefs about scientific evidence pertaining to childhood vaccination—and their beliefs about *what other people think* about the evidence—vary as a function of political ideology. The results of our study suggest that a tentative answer to these questions is yes. At the same time, the purely correlational nature of this research excludes the possibility of drawing any conclusions about causal mechanisms.

Another clear limitation of this study is that the sample was far from statistically representative of the population at large. It would therefore be unwise to assume that the ideological patterns we have observed with respect to beliefs about childhood vaccination would necessarily hold for the United States as a whole, let alone for citizens of other countries. Nevertheless, it may be worth noting that careful comparisons of survey responses provided by MTurk and nationally representative samples suggest that the empirical correlates of political ideology are remarkably similar across these types of samples/platforms and that MTurk seems to be a highly useful tool for the study of political psychology [53]. It is therefore at least conceivable that the results of this study—which is admittedly based on a convenience sample of fewer than 400 participants—will prove useful in planning and interpreting the results of subsequent studies of public opinion based on larger, more statistically representative samples.

From a public health perspective, a few findings from our study may inspire some degree of optimism. To begin with, liberals, moderates, and conservatives in our sample were all more likely to endorse pro- than anti-vaccination statements and to regard more pro- than anti-vaccination statements as “facts” (as opposed to beliefs). Furthermore, there was a significant, albeit modest degree of actual consensus (both within and across ideological groups) that certain statements about childhood vaccination are worthy of endorsement and that they constitute “facts” rather than “beliefs.” In Tables 3 and 4 we have listed five statements that engendered very high and very low levels of agreement, respectively. On one hand, nearly everyone agreed that, “Delaying or refusing vaccinations leaves children unprotected against many dangerous diseases.” On the other hand, there was relatively little consensus about whether “Vaccines introduce toxic chemicals to the body that are not found in the natural immune defenses.”

**Table 3:** Five statements for which there was high agreement concerning whether they should be classified as “facts” and high levels of endorsement as a function of ideological group.

Item	Ideological Group	Percentage of Group Rate as Fact	Percentage of Group Endorse
	Liberals	71.52%	94.94%
“Delaying or refusing vaccinations leaves children unprotected against many dangerous diseases.”	Moderates	68.25%	90.48%
	Conservatives	61.45%	87.95%
	Total	68.12%	91.38%
	Liberals	70.25%	93.67%
“Vaccinations against dangerous diseases have saved more lives than drugs in the late 20th century, such as the development and use of antibiotics.”	Moderates	61.11%	93.65%
	Conservatives	60.24%	83.13%
	Total	64.85%	91.28%
	Liberals	67.72%	94.30%
“Vaccinations are necessary for eliminating vaccine-preventable diseases.”	Moderates	59.52%	93.65%
	Conservatives	55.42%	85.54%
	Total	62.13%	92.10%
	Liberals	81.65%	95.57%
“The creation of vaccinations consists of a long process in order to determine whether or not it is safe and effective for public use.”	Moderates	64.29%	92.06%
	Conservatives	63.86%	84.34%
	Total	71.66%	91.83%
	Liberals	7.59%	10.76%
“The increased number of vaccinations prior to a child’s second birthday is the reason why there has been an increase in Autism Spectrum Disorder in children.”	Moderates	4.76%	21.34%
	Conservatives	6.02%	19.28%
	Total	6.27%	16.35%

**Table 4:** Five statements for which there was low agreement concerning whether they should be classified as “facts” and low levels of endorsement as a function of ideological group.

Item	Ideological Group	Percentage of Group Rate as Fact	Percentage of Group Endorse
	Liberals	26.58%	37.97%
“Vaccines introduce toxic chemicals to the body that are not found in the natural immune defenses.”	Moderates	30.16%	51.59%
	Conservatives	38.55%	50.60%
	Total	30.52%	45.50%
	Liberals	39.24%	46.20%
“Vaccinations do not lead to life-long immunity, whereas contracting a disease, such as chicken pox does result in life-long immunity due to the body’s natural defense mechanisms.”	Moderates	41.27%	53.17%
	Conservatives	43.47%	54.22%
	Total	40.87%	50.41%
	Liberals	22.78%	35.44%
“Vaccinating a child before his/her immune system is fully developed can cause harm to that child.”	Moderates	20.63%	48.41%
	Conservatives	22.89%	38.55%
	Total	22.07%	40.60%
	Liberals	22.78%	32.91%
“Vaccinations can have serious side effects that cause more harm than some of the diseases that they are supposed to prevent.”	Moderates	26.19%	50.79%
	Conservatives	27.71%	40.96%
	Total	25.07%	40.87%
	Liberals	18.35%	25.95%
“The use of aluminum in vaccinations is a risk for Alzheimer’s disease, dementia, and seizures.”	Moderates	21.43%	41.27%
	Conservatives	22.89%	48.19%
	Total	20.43%	36.24%

We did observe ideological divergence with respect to belief in (vs. skepticism about) childhood vaccination and perceived (as opposed to actual) consensus. Specifically, we found that liberals were significantly more likely to endorse pro-vaccination statements and less likely to endorse anti-vaccination statements, in comparison with moderates and conservatives. Furthermore, liberals were significantly more likely to categorize pro-vaccination statements as “facts” and to exhibit within-group consensus about which statements were “facts,” in comparison with moderates and conservatives. Liberals in our convenience sample were also more likely to report vaccinating their children, in comparison with moderates and conservatives. All of these findings are consistent with prior research demonstrating that liberals in the U.S. tend to be more influenced by scientific expertise and empirical evidence in general, compared to other ideological groups [21, 23, 24]. To the extent that it is possible to generalize on the basis of our sample, the findings are inconsistent with the notion that the anti-vaccination movement has taken special hold among those who identify with the political left [17, 18].

We observed a very different type of ideological asymmetry when it came to how accurately people perceived the degree to which others shared their attitudes. Liberals and conservatives assumed that fellow ideologues (and the population at large) would be more likely to share their views than would ideological adversaries. As it turns out, the reality was more complicated. Consistent with prior research [34, 37], conservatives exhibited a “truly false consensus” effect, overestimating the extent to which other conservatives shared their attitudes, whereas liberals exhibited an “illusion of uniqueness,” *underestimating* the extent to which other people shared their attitudes. These differences might well reflect conservatives’ greater desire to share perceptions and opinions with likeminded others [34, 54], as well as liberals’ greater desire to develop a distinctive sense of self that includes holding attitudes that are differentiated from those of others [37]. The implications of these divergent ideological tendencies for health-related beliefs, perceptions, and behaviors would be well worth investigating in subsequent research.

Presumably, ideological disparities arise from the interaction of “top-down” (i.e., sociological) and “bottom-up” (i.e., psychological) factors [16]. That is, liberal-conservative differences in attitudes concerning matters of science and health are probably attributable to a mixture of socially constructed, elite-driven forms of communication and underlying psychological needs. We know from research in political communication that liberals and conservatives tune into different news sources (such as Fox News vs. NPR or MSNBC), and these news sources are likely to highlight contrasting representations of scientific research and health care services; e.g., [55, 56]. Exposure to information stressing the perceived risks of vaccination, for instance, is likely to weaken parental intentions to vaccinate their children [57]. Messages that become familiar through repeated exposure can become highly influential insofar as they are simply assumed to be valid and true [58, 59].

We also know that liberals and conservatives possess different personality profiles, cognitive-motivational styles, and orientations toward uncertainty, threat, and social deviance; e.g., [16, 24]. Theoretically speaking, top-down and bottom-up factors are assumed to be interrelated, insofar as: (a) consistent exposure to certain ideological environments contributes to a mindset that is either open and trusting or skeptical and distrusting of scientific recommendations; and (b) psychological predispositions—including those that reflect differential reactions to uncertainty, threat, and social deviance—shape cultural exposure, including the choice of news and entertainment outlets, as well as interest in scientific information and health education; e.g., [60].

## Conclusions

A great deal of evidence suggests that individuals’ perceptions—and misperceptions—of others’ attitudes and behaviors affect the decisions they make about health-related practices such as smoking [41], alcohol consumption [39], and sexual behavior [42]. Although our study is based on a relatively small, non-representative sample, we have demonstrated that political ideology is linked not only to estimates of the harms and benefits pertaining to childhood vaccination but also to judgments of others’ beliefs and opinions about this important matter. Health-related communications that fail to take into account ideological sources of potential variability in such judgments and estimates are likely to be ineffective; cf. [9]. By pinpointing some of the ways in which liberals, moderates, and conservatives do—and do not—differ when it comes to the perceptions of facts and beliefs and, indeed, the perceptions of social norms, researchers should gain a more nuanced understanding of the structure of public opinion when it comes to childhood vaccination and perhaps other issues as well. In addition, communication specialists will be in a better position to tailor health-related messages to specific audiences so that gaps between popular perceptions and scientific realities may be closed in time.

## Supporting Information

S1 Appendix List of Pro- and Anti-Vaccination Statements. (DOCX)

S2 Appendix Illustration of the calculation of within-subject similarity score. (DOCX)

S3 Appendix Illustration of the calculation of within-subject accuracy score. (DOCX)

## References

[1] Hinman A. Eradication of vaccine-preventable diseases. Annual Review of Public Health. 1999; 20: 211–229. 10352857 10.1146/annurev.publhealth.20.1.211

[2] Kennedy AM, Brown CJ, Gust DA. Vaccine beliefs of parents who oppose compulsory vaccination. Public Health Reports. 2005;120: 252–258. 16134564 10.1177/003335490512000306 PMC1497722

[3] McCoy C. Why are vaccination rates dropping in America? New Republic, 7 24, 2015 Retrieved from: https://newrepublic.com/article/122367/why-are-vaccination-rates-dropping-america

[4] Fox M, Connor T. Think the U.S has a measles problem? Just look at Europe. NBC News 2 7, 2015 Retrieved from: http://www.nbcnews.com/storyline/measles-outbreak/think-u-s-has-measles-problem-just-look-europe-n301726

[5] Sturm LA, Mays RM, Zimet GD. Parental beliefs and decision making about child and adolescent immunization: From polio to sexually transmitted infections. Developmental and Behavioral Pediatrics. 2005;26: 441–452. 10.1097/00004703-200512000-00009 16344662

[6] Gore P, Madhavan S, Curry D, McClung G, Castiglia M, Rosenbluth SA, et al Predictors of childhood immunization completion in a rural population. Social Science & Medicine. 1999;48: 1011–1027. 10390041 10.1016/s0277-9536(98)00410-9

[7] Bates A, Wolinsky FD. Personal, financial, and structural barriers to immunization in socioeconomically disadvantaged urban children. Pediatrics. 1998;101: 591–596. 9521939 10.1542/peds.101.4.591

[8] Funk C, Rainie, L. Attitudes and beliefs on science and technology topics. In Pew Research Center Internet, Science, & Tech Reports. 2015; Available: http://www.pewinternet.org

[9] Betsch C, Sachse K. Dr. Jekyll or Mr. Hyde? (How) the Internet influences vaccination decisions: Recent evidence and tentative guidelines for online vaccine communication. Vaccine. 2012;30: 3723–3726. 10.1016/j.vaccine.2012.03.078 22472790

[10] Waters EA, Weinstein ND, Colditz GA, Emmons K. Explanations for side effect aversion in preventive medical treatment decisions. Health Psychology. 2009;28: 201–209. 10.1037/a0013608 19290712 PMC2657933

[11] Gerend MA, Shepherd JE. Using message framing to promote acceptance of the human papillomavirus vaccine. Health Psychology. 2007;26: 745–752. 18020847 10.1037/0278-6133.26.6.745

[12] Gardner B, Davies A, McAteer J, Michie S. Beliefs underlying UK parents’ views toward MMR promotion interventions: A qualitative study. Psychology, Health, & Medicine. 2010;15: 220–230. 10.1080/13548501003623963 20391239

[13] Ziemer KS, Hoffman M. Beliefs and attitudes regarding human papillomavirus vaccination among college-age women. Journal of Health Psychology. 2013;18: 1360–1370. 10.1177/1359105312462432 23188917

[14] Lewandowsky S, Ecker UKH, Seifert CM, Schwarz N, Cook J. Misinformation and its correction: Continued influence and successful debiasing. Psychological Science in the Public Interest. 2012;13: 106–131. 10.1177/1529100612451018 26173286

[15] Brunson EK. How parents make decisions about their children’s vaccinations. Vaccine. 2013;31: 5466–5470. 10.1016/j.vaccine.2013.08.104 24076175

[16] Jost JT, Federico CM, Napier JL. Political ideologies and their social psychological functions In Freeden M, editor. Oxford handbook of political ideologies. New York: Oxford University Press; 2013 pp. 232–250.

[17] Berezow A, Campbell H. Science left behind: Feel-good fallacies and the rise of the anti-scientific left New York: PublicAffairs; 2012.

[18] Straus I. Mandatory vaccination is conservative and liberals have been the anti-vaccine warriors National Review 2015; Available http://www.nationalreview.com

[19] Berezow A. Are liberals or conservatives more anti-vaccine? RealClearScience Journal Club 2014; Available http://www.realclearscience.com

[20] Gelman A. Red state, blue state, rich state, poor state: Why Americans vote the way they do Expanded ed. Princeton: Princeton University Press; 2009.

[21] Blank JM, Shaw D. Does partisanship shape attitudes toward science and public policy? The case for ideology and religion. The ANNALS of the American Academy of Political and Social Science. 2015;658: 18–35.

[22] Mooney C. The Republican brain: The science of why they deny science–and reality Hoboken: Wiley; 2012.

[23] Kraft PW, Lodge M, Taber CS. Why people ‘don’t trust the evidence’: Motivated reasoning and scientific beliefs. The ANNALS of the American Academy of Political and Social Science. 2014;658: 121–133.

[24] Jost JT, Krochik M. Ideological differences in epistemic motivation: Implications for attitude structure, depth of information processing, susceptibility to persuasion, and stereotyping. Advances in Motivation Science. 2014;1: 181–231.

[25] Mooney C. Vaccination debate flares in GOP presidential race, alarming medical experts Washington Post 2015; Available: http://www.washingtonpost.com

[26] Moore P. Poll results: Vaccinations. You.Gov. 2015; 10: 2 Available https://today.yougov.com/news/2015/02/10/poll-results-vaccination/

[27] Kahan D. Vaccine risk perceptions and ad hoc risk communication: An empirical assessment (CCP Risk Perception Studies No. 17) New Haven: Yale Law School; 2014.

[28] Gettier EL. Is justified true belief knowledge? Analysis. 1963;23: 121–123.

[29] Kuhn TS. The structure of scientific revolutions Chicago: University of Chicago Press; 1962.

[30] Goodwin GP, Darley JM. Why are some moral beliefs perceived to be more objective than others? Journal of Experimental Social Psychology. 2012;48: 250–256.

[31] Marsh EJ, Meade ML, Roediger HL. (2003). Learning facts from fiction. Journal of Memory and Language. 2013;49: 519–536.

[32] Rabinowitz M, Acevedo M, Casen S, Rosengarten M, Kowalczyk M, Blau Portnoy L. Distinguishing facts from beliefs: Fuzzy categories. Psychology of Language and Communication. 2013;17: 241–267

[33] Festinger L, Schachter S, Back K. Social pressure in informal groups Stanford: Stanford University Press; 1950.

[34] Stern C, West TV, Jost JT, Rule NO. “Ditto heads” Do conservatives perceive greater consensus within their ranks than liberals? Personality and Social Psychology Bulletin. 2014;40: 1162–1177. 24972941 10.1177/0146167214537834

[35] Gvirsman SD. Size matters: The effect of political orientation, majority status, and majority size on misconceptions of public opinion. Public Opinion Quarterly. 2015;79: 1–27.

[36] Krueger J, Clement RW. The truly false consensus effect: An ineradicable and egocentric bias in social perception. Journal of Personality and Social Psychology. 1994;68: 579–610. 10.1037//0022-3514.67.4.596 7965607

[37] Stern C, West TV, Schmitt PG. The liberal illusion of uniqueness. Psychological Science. 2014;25: 137–144. 10.1177/0956797613500796 24247730

[38] Cialdini RB. Influence: The psychology of persuasion New York: Collins; 2007.

[39] Prentice DA, Miller DT. Pluralistic ignorance and alcohol use on campus: Some consequences of misperceiving the social norm. Journal of Personality and Social Psychology. 1993;64: 243–256. 8433272 10.1037//0022-3514.64.2.243

[40] Prinstein M, Wang S. False consensus and adolescent peer contagion: Examining discrepancies between perceptions and actual reported levels of friends’ deviant and health risk behaviors. Journal of Abnormal Child Psychology. 2005;33: 293–306. 15957558 10.1007/s10802-005-3566-4

[41] Sherman SJ, Presson CC, Chassin L, Corty E, Olshavsky R. The false consensus effect in estimates of smoking prevalence underlying mechanisms. Personality and Social Psychology Bulletin. 1983;9: 197–207.

[42] Suls J, Wan CK, Sanders GS. False consensus and false uniqueness in estimating the prevalence of health-protective behaviors. Journal of Applied Social Psychology. 2006;18: 66–79.

[43] Buhrmester M, Kwang T, Gosling SD. Amazon’s Mechanical Turk: A new source of inexpensive, yet high-quality, data? Perspectives on Psychological Science. 2011;6: 3–5. 10.1177/1745691610393980 26162106

[44] Fox WS, Williams JD. Political orientation and music preferences among college students. Public Opinion Quarterly. 1974;38: 352–371.

[45] Fitzmaurice G, Laird N, Ware J. Applied longitudinal analysis 2^nd^ ed. Hoboken: John Wiley & Sons, Inc; 2011.

[46] Aiken LS, West SG. Multiple regression: Testing and interpreting interactions Newbury Park: Sage; 1991.

[47] Liang K, Zeger SL. Longitudinal data analysis using generalized linear models. Biometrika. 1986;73: 13–22.

[48] Kenny DA, La Voie L. The social relations model In Berkowitz L, editor. Advances in experimental social psychology. Volume 19 New York: Academic Press; 1984 pp. 141–182.

[49] Kenny DA. Interpersonal perception: A social relations analysis New York: Guilford Press; 1994. 3317468

[50] Krueger J, Zeiger JS. Social categorization and the truly false consensus effect. Journal of Personality and Social Psychology. 1993;65: 670–680.

[51] McNemar Q. Psychological statistics (3rd edition). New York: Wiley; 1962.

[52] Cohen BH. Explaining psychological statistics (4th edition). New York: Wiley; 2013.

[53] Clifford S, Jewell RM, Waggoner PD. Are samples drawn from Mechanical Turk valid for research on political ideology? Research and Politics. 2015; Oct-Dec: 1–9

[54] Jost JT, Ledgerwood A, Hardin CD. Shared reality, system justification, and the relational basis of ideological beliefs. Social and Personality Psychology Compass. 2008;2: 171–186.

[55] Iyengar S, Hahn KS. Red media, blue media: Evidence of ideological selectivity in media use. Journal of Communication. 2009;59: 19–39

[56] Levendusky M. How partisan media polarize America Chicago: University of Chicago Press; 2013.

[57] Betsch C, Renkewitz F, Betsch T, Ulshöfer C. (2010). The influence of vaccine-critical Internet pages on perception of vaccination risks. Journal of Health Psychology. 2010;15: 446–455. 10.1177/1359105309353647 20348365

[58] Ecker UKH, Swire B, Lewandowsky S. Correcting misinformation–A challenge for education and cognitive science In Rapp DN, Braasch J, editors. Processing inaccurate information: Theoretical and applied perspectives from cognitive science and the educational sciences. Cambridge: MIT Press; 2014 pp. 13–38.

[59] Schwarz N, Sanna LJ, Skurnik I, Yoon C. Metacognitive experiences and the intricacies of setting people straight: Implications for debiasing and public information campaigns. Advances in Experimental Social Psychology. 2007;39: 127–161.

[60] Xu X, Mar RA, Peterson J. Does cultural exposure partially explain the association between personality and political orientation? Personality and Social Psychology Bulletin. 2013;39: 1497–1517. 10.1177/0146167213499235 23928399

