# Supplementary material for: Beliefs about Childhood Vaccination in the United States: Political Ideology, False Consensus, and the Illusion of Uniqueness
Source: PLoS One. 2016 Jul 8;11(7):e0158382. doi: 10.1371/journal.pone.0158382 (PMC4938547; doi:10.1371/journal.pone.0158382)
Supplement: S1 Appendix — (DOCX) [file pone.0158382.s001.docx]

**Appendix A - List of Pro- and Anti-Vaccination Statements**

**Pro-Vaccination Statements**

1. Eighty to 90% of a population needs to be vaccinated in order for an entire community to be fully protected against a disease.

2. Delaying or refusing vaccinations leaves children unprotected against many dangerous diseases.

3. Vaccinations against dangerous diseases have saved more lives than drugs in the late 20th century, such as the development and use of antibiotics.

4. The amount of ingredients used to create vaccinations is safe.

5. It is very rare to have an adverse reaction to a vaccine.

6. Mothers who are vaccinated protect their unborn children from viruses that can cause birth defects, such as mental disabilities, heart problems, and hearing and vision loss.

7. Vaccinations are necessary for eliminating vaccine-preventable diseases.

8. Vaccinations mobilize antibodies and proteins that are mimicked in the body’s natural immune defenses.

9. The creation of vaccinations consists of a long process in order to determine whether or not it is safe and effective for public use.

10. There is no direct link between vaccinations and Autism Spectrum Disorder or other mental disabilities.

**Anti-Vaccination Statements**

11. The increased number of vaccinations prior to a child’s second birthday is the reason why there has been an increase in Autism Spectrum Disorder in children.

12. Vaccinations expose children to mercury through the use of thimerosal, which is a reason for the rise in Autism Spectrum Disorder.

13. Vaccinations can have serious side effects that cause more harm than some of the diseases that they are supposed to prevent.

14. Vaccines introduce toxic chemicals to the body that are not found in the natural immune defenses.

15. Even if people are vaccinated, there is still a risk of contracting the disease that the vaccination was intended to protect against.

16. Vaccinating a child before his/her immune system is fully developed can cause harm to that child.

17. An increase in hygiene and improved living conditions are the reasons why the prevalence of diseases have declined; it is not a result of an increase in vaccinations.

18. The use of aluminum in vaccinations is a risk for Alzheimer’s disease, dementia, and seizures.

19. Vaccinations do not lead to life-long immunity, whereas contracting a disease, such as chicken pox does result in life-long immunity due to the body’s natural defense mechanisms.

20. Due to political ties and economic incentives, pharmaceutical companies are not trustworthy sources to regulate the safety of vaccinations.
