# Supplementary material for: Beliefs about Childhood Vaccination in the United States: Political Ideology, False Consensus, and the Illusion of Uniqueness
Source: PLoS One. 2016 Jul 8;11(7):e0158382. doi: 10.1371/journal.pone.0158382 (PMC4938547; doi:10.1371/journal.pone.0158382)
Supplement: S2 Appendix — (DOCX) [file pone.0158382.s002.docx]

**Appendix B – Illustration of the calculation of within-subject similarity score.**

To illustrate the calculation of within-subject assumed similarity scores, we will use data for five items from a hypothetical participant, as shown in the table below. We correlated the participant’s own opinions (Column A) with their estimates of the percentage of others who agreed with each item (Column B). This method results in a single assumed similarity score for each participant (see also Krueger & Zeiger, 1993, for further discussion of this analytic strategy).

| Item | Opinion (A) | Perceived Agreement (B) |
| --- | --- | --- |
| 1 | 3 | 30 |
| 2 | 1 | 52 |
| 3 | 4 | 87 |
| 4 | 2 | 45 |
| 5 | 2 | 60 |
