# Supplementary material for: Beliefs about Childhood Vaccination in the United States: Political Ideology, False Consensus, and the Illusion of Uniqueness
Source: PLoS One. 2016 Jul 8;11(7):e0158382. doi: 10.1371/journal.pone.0158382 (PMC4938547; doi:10.1371/journal.pone.0158382)
Supplement: S3 Appendix — (DOCX) [file pone.0158382.s003.docx]

**Appendix C – Illustration of the calculation of within-subject accuracy score.**

To illustrate the calculation of within-subject accuracy scores, we will use data from a hypothetical participant, as shown in the table below. We first calculated the percentage of people who actually agreed with each item (Column C). To calculate this value for each item we dichotomized attitudes toward the items, such that responses of 1 and 2 were categorized as disagreement with the item and responses of 3 and 4 were categorized as agreement. We then calculated a discrepancy score for each item (Column D). To create this discrepancy score, we subtracted the percentage of people who agreed with an item (Column C) from the participant’s estimate of the number of people who agreed with the item (Column B). We then correlated the perceiver’s opinions (Column A) with the discrepancy scores (Column D). This method results in a single accuracy correlation for each participant.

| Item | Opinion (A) | Perceived Agreement (B) | Actual Agreement (C) | Perceived Agreement – Actual Agreement (D) |
| --- | --- | --- | --- | --- |
| 1 | 3 | 45 | 60 | -15 |
| 2 | 4 | 60 | 85 | -25 |
| 3 | 2 | 25 | 30 | -5 |
| 4 | 4 | 70 | 50 | 20 |
| 5 | 1 | 55 | 70 | -15 |
